# A Case of Ophthalmoplegia, Hypotonia, and Developmental Delay in the Setting of Corpus Callosum Hypoplasia

**DOI:** 10.7759/cureus.25930

**Published:** 2022-06-14

**Authors:** Erica Y Kim, Sergio Trejo, Eric B Nguyen, Michelle I Malwane, José R Cucalón-Calderón

**Affiliations:** 1 Pediatrics, University of Nevada Reno School of Medicine, Reno, USA

**Keywords:** agenesis of the corpus callosum, hypotonia, developmental delay, corpus callosum hypoplasia, strabismus, ophthalmoplegia

## Abstract

Anomalies of the corpus callosum, including complete agenesis, partial agenesis, and hypoplasia, are some of the most common brain malformations. Corpus callosum abnormalities are potentially syndromic, many of which have identifiable genetic etiologies. Patients affected with either syndromic or non-syndromic corpus callosum anomalies may also have associated ophthalmologic abnormalities. Some of the syndromes with corpus callosum malformations that also involve ophthalmologic findings include Aicardi syndrome, Mowat-Wilson syndrome, and Xia-Gibbs syndrome. This case report describes a patient with hypoplasia and possible dysgenesis of the corpus callosum noted on magnetic resonance imaging (MRI) who had several ophthalmologic findings, including ophthalmoplegia, strabismus, and nystagmus, associated with microcephaly, dysmorphic facial features, global developmental delay, hypotonia, and cryptorchidism. While several previously identified syndromes share similar clinical features with this patient, these findings may also represent an unidentified genetic syndrome, and the patient remains under evaluation for a genetic diagnosis. This report explores the differential for ophthalmologic abnormalities in the setting of corpus callosum hypoplasia.

## Introduction

Corpus callosum anomalies, including complete agenesis, partial agenesis (dysgenesis), and hypoplasia, are among the most common brain malformations. A recent population-based study reported a prevalence of 2.49 per 10,000 births in the general population [1] and a prevalence of two to three percent in children with developmental disabilities [2]. Studies have shown that approximately 30%-40% of cases of agenesis of the corpus callosum have an identifiable cause, with about 10% being associated with chromosomal anomalies such as trisomy 18 and 13 and about 20%-30% being associated with gene mutations and syndromes, including Aicardi syndrome, Sotos syndrome, and acrocallosal syndrome [3,4]. Genetic factors, prenatal infections, and maternal alcohol use are associated with anomalies of the corpus callosum due to the disruption of callosal development [5]. Corpus callosum anomalies may be present as an isolated abnormality but can also occur with other central nervous system malformations, including Chiari malformation, schizencephaly, colpocephaly, and polymicrogyria [6].

The visual system can be affected in patients with corpus callosum abnormalities. Two studies that evaluated for ocular abnormalities in patients with anomalies of the corpus callosum, excluding those with Aicardi syndrome, found decreased visual acuity, refractive errors, strabismus, optic atrophy, and nystagmus to be some of the ocular pathologies present [7,8]. These findings were seen in both syndromic and non-syndromic patients. Out of the over 200 genetic syndromes with corpus callosum malformations that have been described, several include ocular abnormalities such as Aicardi syndrome; Mowat-Wilson syndrome; agenesis of corpus callosum, cardiac, ocular, and genital syndrome (ACOGS); Xia-Gibbs syndrome; and congenital fibrosis of the extraocular muscles type three (CFEOM3).

This case report describes a patient with ocular findings, including ophthalmoplegia, strabismus, and nystagmus, associated with dysmorphic facial features, microcephaly, developmental delay, hypotonia, and bilateral cryptorchidism. Magnetic resonance imaging (MRI) of the brain revealed hypoplasia and possible dysgenesis of the corpus callosum. This patient presentation may represent a genetic disorder that has not yet been identified, and this report highlights the differential for ophthalmologic abnormalities in the setting of corpus callosum hypoplasia.

## Case presentation

A two-week-old male was brought to a primary care pediatrician for a well-child visit. Upon examination, the child appeared to have low-set ears, broad nasal bridge, bulbous nose, thin upper lip, and mild retrognathia. His head circumference was 33 cm, less than the first percentile for his age. The patient had a normal birth history, as he was born at a gestational age of 38 weeks five days to a gravida two para one mother via normal spontaneous vaginal delivery. The mother reported no alcohol or drug use during pregnancy. All prenatal labs and prenatal ultrasounds were normal. The newborn examination did not reveal any abnormalities at the time, and newborn screenings showed normal results.

The patient returned for his two- and four-month well-child checks, at which the pediatrician noted new abnormalities on eye examination. The patient displayed bilateral fixed exotropia and horizontal nystagmus. Though retrognathia and low-set ears were no longer evident, some of the other facial anomalies previously noted were still present, including the broad nasal bridge and thin upper lip. The patient also had global developmental delay, as he was unable to roll from stomach to back or keep his chest up when prone, did not have a social smile, did not laugh aloud, and had poor visual tracking. The patient’s length at four months was 0.597 m (second percentile), and weight was 5.2 kg (less than the first percentile), indicating failure to thrive. The patient also had microcephaly, as his head circumference was 38.7 cm (less than the first percentile). The physical exam also revealed generalized hypotonia with head lag greater than 60 degrees, as well as bilateral cryptorchidism. Given these findings, referrals were placed for pediatric neurology, ophthalmology, and genetics. The patient was also referred to early intervention services and was evaluated by a physical therapist and occupational therapist for developmental delay and hypotonia, as well as a speech-language pathologist for potential feeding difficulties associated with hypotonia.

The patient was seen by all the specialists mentioned above. The neurologist performed an electroencephalogram when the patient was three months old that showed normal results. The neuro-ophthalmologist, who evaluated the patient, described the patient’s complex eye motility disorder as ophthalmoplegia. On ophthalmologic examination, the patient was noted to fix but not follow and had a large exotropia with limitation to elevation and adduction, mild limitation to abduction, and mild bilateral globe retraction. Given these limitations, the patient would use jerking and thrusting movements of his head to look around. The oculocephalic reflex maneuver elicited abducting nystagmus. Fundus exam revealed an anomalous appearing optic nerve on the right and myelinated nerve fibers on the left. The specialists all recommended an MRI of the brain and orbits as well as genetic testing, and the ophthalmologist recommended strabismus surgery due to the degree of exotropia.

**Figure 1:**
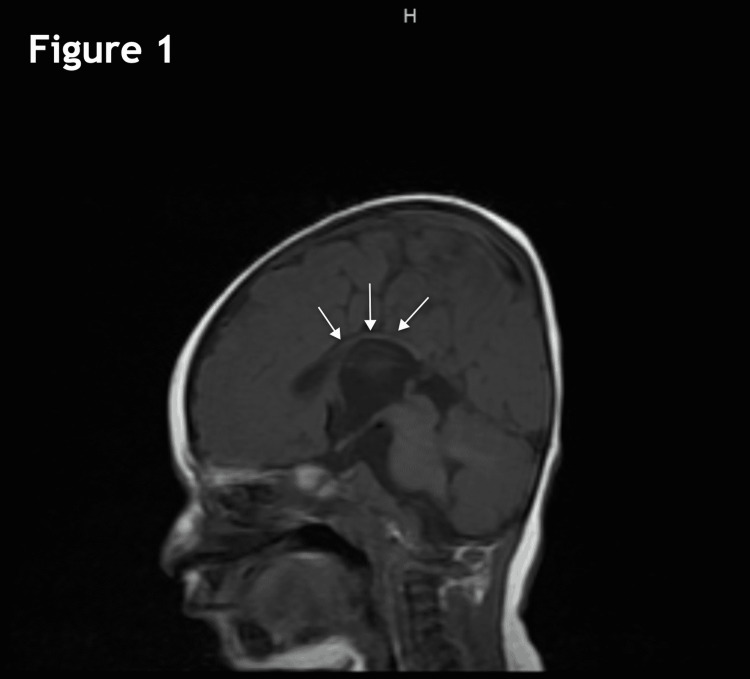
Sagittal T1-weighted image showing marked thinning of the corpus callosum with possible partial dysgenesis

**Figure 2:**
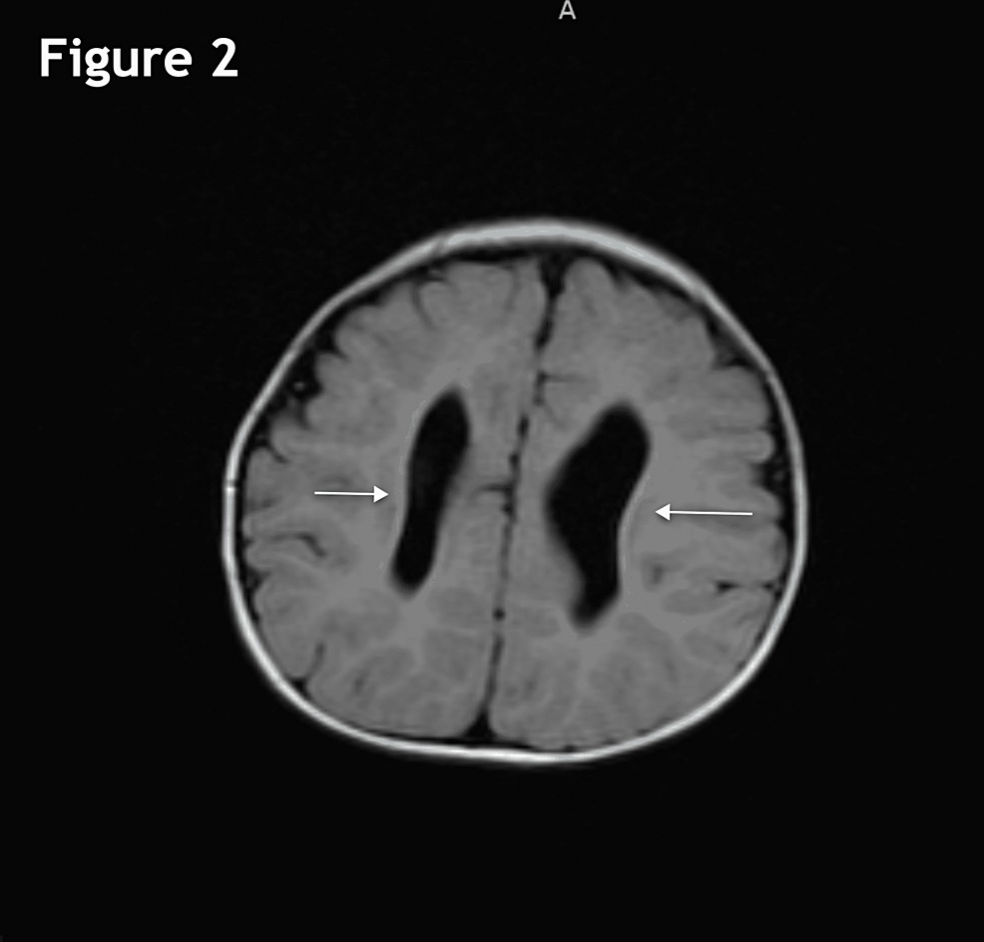
Axial flair image showing the parallel orientation of the lateral ventricles and mild lateral ventriculomegaly

**Figure 3:**
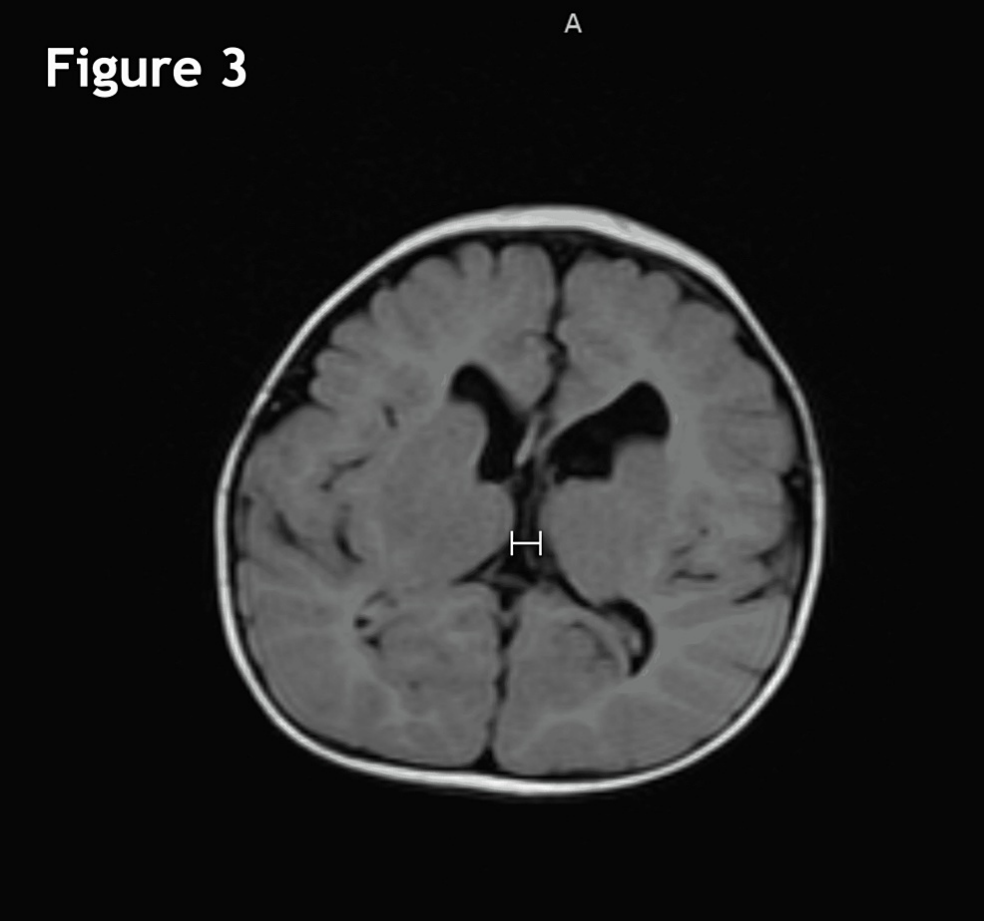
Axial flair image showing mild ventriculomegaly of the third ventricle

The MRI performed at four months of age revealed severe thinning of the corpus callosum with possible partial dysgenesis (Figure 1), the parallel orientation of the lateral ventricles (Figure 2), and mild ventriculomegaly of the lateral and third ventricles (Figures 2, 3). Other workups at this time included SNP microarray, which did not detect any clinically significant copy number changes or regions of homozygosity. Serum amino acid profile, creatine kinase, TSH, and free T4 were all normal as well. A high-resolution karyotype was unable to be performed due to a lack of approval from insurance.

**Figure 4:**
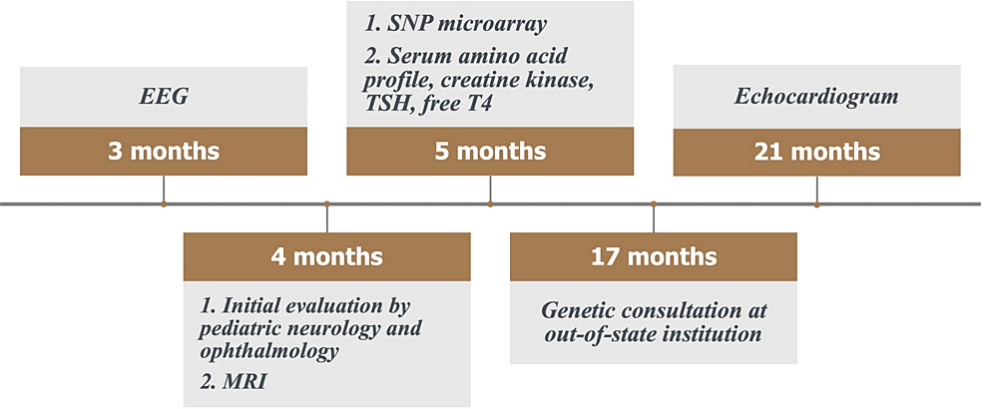
Timeline of diagnostic workup with patient age in months

As care continued, the ophthalmologist performed a bilateral lateral rectus recession at 12 months of age, which improved the exotropia but did not resolve it completely. The patient’s development remained delayed, with the patient starting to smile and roll over in both directions at seven months, prop sit at 10 months, army crawl at 12 months, cruise at 15 months, and walk with one hand support at 18 months. At 17 months, the patient was seen at an out-of-state institution by a geneticist (Figure 4). Due to COVID-19 restrictions, the patient was unable to be seen in person and was evaluated through a video visit, which the geneticist stated limited the ability to properly assess the patient and develop a plan. Further assessments recommended included an echocardiogram to assess for cardiac anomalies, which were normal, as well as whole-exome sequencing, which has not yet been performed. Throughout the patient’s care, he has been seen regularly by a physical therapist, occupational therapist, and speech-language pathologist. No pharmacologic interventions have been used for this patient, as none were recommended by the specialists. At this time, an in-person visit with the geneticist is being pursued, and the patient continues to be evaluated for a possible genetic diagnosis.

## Discussion

This case report describes a patient with corpus callosum hypoplasia who has several ocular findings, including ophthalmoplegia, strabismus, nystagmus, and myelinated nerve fiber layer, as well as dysmorphic facial features, microcephaly, developmental delay, hypotonia, and cryptorchidism. This presentation is likely syndromic and due to a genetic abnormality, but the cause has not yet been identified in this patient, as the microarray was normal and whole-exome sequencing has not yet been performed. Over 200 syndromes have been described that involve abnormalities of the corpus callosum, and this patient’s presentation may be confirmed to fall into one of these categories once further genetic testing is performed.

Of particular interest, in this case, are the ocular findings in this patient. There are several syndromes and genetic disorders involving abnormalities of the corpus callosum that also present with ocular abnormalities similar to the patient described in this case. For example, Mowat-Wilson syndrome, an autosomal dominant disorder that occurs due to de novo heterozygous mutations in the *ZEB2 *gene, can involve hypoplasia or agenesis of the corpus callosum and various ocular findings such as strabismus and nystagmus. It is characterized by distinct facial features such as broad nasal bridge, broad eyebrows, hypertelorism, pointed chin, and uplifted earlobes, and involves developmental delay and intellectual disability. It can also include microcephaly, short stature, hypotonia, seizures, Hirschsprung disease or chronic constipation, congenital heart defects, and genitourinary anomalies such as hypospadias and cryptorchidism [9,10]. The patient, in this case, has several of these features, and therefore Mowat-Wilson is a possibility.

Another syndrome with some of the features seen in this patient case is ACOGS, which is caused by a heterozygous mutation in the *CDH2* gene and has been associated with corpus callosum agenesis or hypoplasia, developmental delay and intellectual disability, ocular findings, including strabismus and Duane anomaly, congenital heart defects, cryptorchidism, and craniofacial dysmorphisms such as a thin upper lip, hypertelorism, flat nasal bridge, and low-set ears [11]. Additionally, the first reports of Xia-Gibbs syndrome, caused by a heterozygous mutation in the *AHDC1* gene, involved children with corpus callosum hypoplasia, intellectual disability, delayed development, failure to thrive, hypotonia, esotropia, dysmorphic features, including low-set ears and flat nasal bridge, and obstructive sleep apnea [12].

Another genetic disorder to be considered in this case is congenital fibrosis of the extraocular muscles (CFEOM), which has both autosomal dominant and autosomal recessive variants. It is characterized by non-progressive ophthalmoplegia, with or without ptosis, that occurs due to abnormal oculomotor nerve development that leads to hypoplasia of the extraocular muscles innervated by this nerve [13]. These patients present with limitations of vertical gaze and potentially of horizontal gaze, and compensate for these limitations by moving their head to track objects. Strabismus is present as well. One subtype, CFEOM3, has been associated with brain malformations, including hypoplasia or agenesis of the corpus callosum, as well as polymicrogyria, schizencephaly, and dysgenesis of the olfactory bulbs [14].

Toriello-Carey syndrome and genitopatellar syndrome are two conditions that also may be relevant to this case. While they are not commonly associated with functional ophthalmologic abnormalities, they both involve agenesis of the corpus callosum and share some other clinical features with the patient described in this case report. Toriello-Carey syndrome involves mental retardation, hypotonia, growth delay, Pierre Robin sequence, cardiac defects, genital anomalies, and characteristic facial features, including wide-spaced eyes, flattened nasal bridge, and short neck [15]. The genetic etiology of Toriello-Carey syndrome is unclear as there have been no candidate genes or chromosomal anomalies that have been identified as the cause, but the inheritance pattern of Toriello-Carey syndrome is thought to be autosomal recessive. Genitopatellar syndrome is caused by a heterozygous mutation in the *KAT6B* gene and is characterized by patellar hypoplasia or agenesis, flexion contractures of the hips and knees, urogenital anomalies, developmental delay, hypotonia, and facial anomalies, including low-set ears and thin upper lip [16].

While ocular findings can be associated with genetic disorders, it is relevant to note that nonsyndromic patients with corpus callosum malformations can have ocular findings similar to patients with a genetic disorder or identified syndrome [7]. In addition, patients with corpus callosum anomalies are likely to have other CNS malformations present as well. These factors make it difficult to assess whether certain ocular abnormalities seen in patients with corpus callosum anomalies are mere associations or if there is some type of causative effect, especially considering the role of the corpus callosum in the maturation of the visual cortex and its role in visual perception [17,18].

In order to determine the diagnosis for this patient, he will need further genetic testing, such as whole-exome sequencing. Whole exome sequencing, in this case, may be limited by the fact that trio testing to help identify de novo variants would be difficult given the lack of contact with the patient’s father. Targeted gene testing, such as testing for the *ZEB2* gene affected in Mowat-Wilson syndrome, maybe a reasonable next step as well. Barriers to further testing include the cost of these tests, limited access to genetic services within the state, and public insurance coverage under Medicaid limiting where services can be provided. Of note, the patient’s family identifies as Latinx/Hispanic and is Spanish-speaking only, which poses additional language and cultural barriers.

## Conclusions

Ophthalmologic abnormalities have been shown to be associated with anomalies of the corpus callosum, both in syndromic and non-syndromic patients. Some of these abnormalities include strabismus, nystagmus, optic atrophy, and decreased visual acuity. This case report describes a child with ocular findings, including ophthalmoplegia, strabismus, and nystagmus, associated with dysmorphic facial features, global developmental delay, hypotonia, and bilateral cryptorchidism who was found to have hypoplasia and possible dysgenesis of the corpus callosum on MRI. Several syndromes that involve corpus callosum anomalies and ophthalmologic findings that also share other common features with this patient include Mowat-Wilson syndrome, ACOGS, Xia-Gibbs syndrome, and CFEOM3. While the SNP microarray was negative, further genetic testing may provide a genetic explanation for the patient’s presentation. Targeted gene testing and whole exome sequencing are potential next steps, but barriers to further evaluation and testing exist, including the cost of testing and specialty provider visits, difficulties with insurance coverage under Medicaid, and limited access to genetic services locally.

## References

[1] Ballardini E, Marino P, Maietti E, Astolfi G, Neville AJ (2018). Prevalence and associated factors for agenesis of corpus callosum in Emilia Romagna (1981-2015). Eur J Med Genet.

[2] Jeret JS, Serur D, Wisniewski K, Fisch C (1985). Frequency of agenesis of the corpus callosum in the developmentally disabled population as determined by computerized tomography. Pediatr Neurosci.

[3] Schaefer GB, Bodensteiner JB, Buehler BA (1997). The neuroimaging findings in Sotos syndrome. Am J Med Genet A.

[4] Courtens W, Vamos E, Christophe C (1997). Acrocallosal syndrome in Algerian boy born to consanguineous parents: Review of the literature and further delineation of the syndrome. Am J Med Genet A.

[5] Riley EP, Mattson SN, Sowell ER, Jernigan TL, Sobel DF, Jones KL (1995). Abnormalities of the corpus callosum in children prenatally exposed to alcohol. Alcohol Clin Exp Res.

[6] Bedeschi MF, Bonaglia MC, Grasso R (2006). Agenesis of the corpus callosum: clinical and genetic study in 63 young patients. Pediatr Neurol.

[7] Kızıltunç PB, Şahlı E, İdil A, Atilla H (2021). Demographic, ocular and associated neurological findings in corpus callosum malformations. Turk J Pediatr.

[8] Goyal R, Watts P, Hourihan M (2010). Ocular findings in pediatric patients with partial agenesis of corpus callosum. J Pediatr Ophthalmol Strabismus.

[9] Adam MP, Conta J, Bean LJH (2007). Mowat-Wilson Syndrome. GeneReviews [Internet].

[10] Adam MP, Schelley S, Gallagher R (2006). Clinical features and management issues in Mowat-Wilson syndrome. Am J Med Genet A.

[11] Accogli A, Calabretta S, St-Onge J (2019). De novo pathogenic variants in N-cadherin cause a syndromic neurodevelopmental disorder with corpus callosum, axon, cardiac, ocular, and genital defects. Am J Hum Genet.

[12] Xia F, Bainbridge MN, Tan TY (2014). De novo truncating mutations in AHDC1 in individuals with syndromic expressive language delay, hypotonia, and sleep apnea. Am J Hum Genet.

[13] Whitman MC, Engle EC (2017). Ocular congenital cranial dysinnervation disorders (CCDDs): insights into axon growth and guidance. Hum Mol Genet.

[14] Demer JL, Clark RA, Tischfield MA, Engle EC (2010). Evidence of an asymmetrical endophenotype in congenital fibrosis of extraocular muscles type 3 resulting from TUBB3 mutations. Invest Ophthalmol Vis Sci.

[15] Toriello HV, Colley C, Bamshad M (2016). Update on the Toriello-Carey syndrome. Am J Med Genet A.

[16] Zhang LX, Lemire G, Gonzaga-Jauregui C (2020). Further delineation of the clinical spectrum of KAT6B disorders and allelic series of pathogenic variants. Genet Med.

[17] Pietrasanta M, Restani L, Caleo M (2012). The corpus callosum and the visual cortex: plasticity is a game for two. Neural Plast.

[18] Bui Quoc E, Ribot J, Quenech'du N (2011). Asymmetrical interhemispheric connections develop in cat visual cortex after early unilateral convergent strabismus: anatomy, physiology, and mechanisms. Front Neuroanat.

